# Supplementary material for: Adaptive tactile interaction transfer via digitally embroidered smart gloves
Source: Nat Commun. 2024 Jan 29;15:868. doi: 10.1038/s41467-024-45059-8 (PMC10825181; doi:10.1038/s41467-024-45059-8)
Supplement: Supplementary file 1 — Supplementary Information [file 41467_2024_45059_MOESM1_ESM.pdf]

Supplementary Materials for Adaptive Tactile Interaction Transfer via Digitally Embroidered Smart Gloves

Yiyue Luo[1*], Chao Liu[1], Young Joong Lee[1], Joseph DelPreto[1], Kui Wu[2], Michael Foshey[1], Daniela Rus[1], Tomás Palacios[1], Yunzhu Li[3], Antonio Torralba[1] and Wojciech Matusik[1*]

[1*]Department of Electrical Engineering and Computer Science, Massachusetts Institute of Technology, 32 Vassar St., Cambridge, 02139, MA, USA.
[2]LightSpeed Studios, 12777 W Jefferson Blvd., Los Angeles, 90066, CA, USA.
[3]Department of Computer Science, University of Illinois Urbana-Champaign, 201 North Goodwin Avenue, Urbana, 61801, IL, USA.

*Corresponding author(s). E-mail(s): yiyueluo@mit.edu; wojciech@mit.edu;
Contributing authors: chaoliu@csail.mit.edu; youngyjl@mit.edu; delpreto@csail.mit.edu; kwwu@global.tencent.com; mfoshey@mit.edu; rus@csail.mit.edu; tpalacios@mit.edu; yunzhuli@illinois.edu; torralba@mit.edu;

This PDF file includes:

Supplementary Notes
Supplementary Figure 1-15

Other Supplementary Materials include the following:

Supplementary Movie 1 to 7
Supplementary Data 1

Supplementary Notes

Mechanical testing on embroidered coils

We conducted tensile tests on pure non-woven fabric, pure Spandex fabric, and fabric that incorporated embroidered enameled copper wire. The tests were conducted on 20 cm samples using a Shimadzu AGS-X mechanical tester, operating at a speed of 20 mm/min. The pure non-woven fabric substrate exhibits a tensile strength of approximately 28 N, with a maximum elongation of 75%. When non-woven fabric is embroidered with enameled copper wire, the sample maintains a similar Young's modulus but breaks at a maximum elongation of 15%, primarily due to the enameled copper wire's fracture, as depicted in Supplementary Fig.3b. On the other hand, the pure Spandex fabric enables maximum elongation of up to 180%. Spandex with embroidered copper wires breaks at a maximum elongation of 25%, with greater elasticity and lower Young's modulus (as evident in the light blue curve in Supplementary Fig.3a. We speculate that the increased elongation is attributable to the smoother surface of Spandex fabric, resulting in reduced friction and tolerating greater movement of the enameled copper wire during stretching. A glove typically endures elongation in the range of 10%-20% during daily activities; therefore, it is reasonable to assume that our smart gloves can withstand stretching during the bending and movement of our hands.

We further investigated the durability of non-woven fabric embroidered with enameled copper wire by subjecting a 20 cm sample to 3000 bending cycles. As demonstrated in the following Supplementary Fig.3c, the bending cycles were performed in a tensile test setup, where the sample was placed at the initial positions with a minimum bending radius of 3.18 cm. It was then stretched with a tensile length of 10 cm. The following Supplementary Fig.3d shows photos of the embroidered vibrotactile coils at 0, 1000, 2000, and 3000 cycles. Notably, no significant changes were observed. Throughout our user studies and experiments, our gloves have endured over 6 hours of use, involving more than 100 instances of wearing and removal, with no noticeable performance degradation in the tactile sensors and vibrotactile haptic units.

Customized design pipeline

We enable rapid and customized design and fabrication of the gloves (Supplementary Fig.2). We first take a picture of a person's hand with a fixed reference (i). We then extract key parameters on the size of the hands from the pictures, such as the length of fingers, the width of the palm, and so on (ii). Such parameters were then able to be mapped to a pre-defined glove design template in any vector-based interactive user interface, e.g. Adobe Illustrator, Inkscape, etc (iii). Here we fixed the arrangement of the placement of tactile sensors and vibrotactile haptics albeit their placement and design parameters can be easily customized for users' needs as well. The customized glove designs were then converted into .dst files containing both the vibrotactile coils designs

and the glove outlines (iv), and were automatically fabricated via the digital embroidery machine (v).

In future work, we would like to develop a more complex computation design pipeline that incorporates an interactive user interface and automatic parameter fine-tuning.

Integrated magnets

Our integrated permanent magnets are comparable in size to rigid buttons and zippers commonly found on garments (6.35 mm in radius and 1.6 mm in thickness for all application scenarios). We therefore consider our system a textile-based wearable interface. Each permanent magnet weighs 0.377 g and the enameled copper wire weighs less than 1 g per meter. In our full-sized smart glove designs, the 23 vibrotactile haptic units and connecting traces add less than 15 g to the original glove. For reference, the plain spandex-based glove weighs around 10 g, and the plain commercial thick handling glove for the tactile interaction transfer demonstration weighs 200 g.

For future work, we propose substituting permanent magnets with soft magnets so that our system can be entirely soft, albeit resulting in weaker magnetic fields.

Time delay of the system

In general, we consider that the tactile sensors transmit signal and the vibrotactile haptic actuators output response with negligible time delay. More specifically, the tactile sensing data was serialized to a laptop at the frame rate of 60 Hz, with a time delay of 16 ms. The model takes less than 1 ms to output the optimized haptic sequence with the use of NVIDIA RTX 3080. The vibrotactile haptic actuators are controlled by Arduino Mega 2560 Rev3 with a clock speed of up to 16 MHz. The microcontroller receives and executes actuating signals at each digital pin with a baud rate of 115200 bits per second. The execution of the vibrotactile actuators is controlled by an array of H-bridges consisting of N-MOSFETs (IRLB8721PbF, Infineon Technologies). According to the spreadsheet and as demonstrated in the following figure, during switching, the turn-on delay time ($t_{d(on)}$) is 9 ns, the turn-off delay time ($t_{d(off)}$) is 9 ns, and the rise time (t_r) and fall time (t_f) are 91 ns and 17 ns respectively. For the teleoperation application specifically, the delay also comes from the capturing of users' finger position via webcam and the estimation of the distance between fingers, which is around 20 Hz, with a time delay of 50 ms, as well as the transmission of distance information to the robot gripper controller, which is at 50 Hz.

Overall, the time delay from the capturing of tactile signal, and the optimization, transmission, and execution of the vibrotactile haptic sequences is less than 30 ms. There will be a perception delay, i.e., the time lag between the haptic signal output and the user's actual actions. Such delay is inevitable

and our goal is to optimize the haptic output to minimize the effect of such delay on the users' performance.

Supplementary Figure

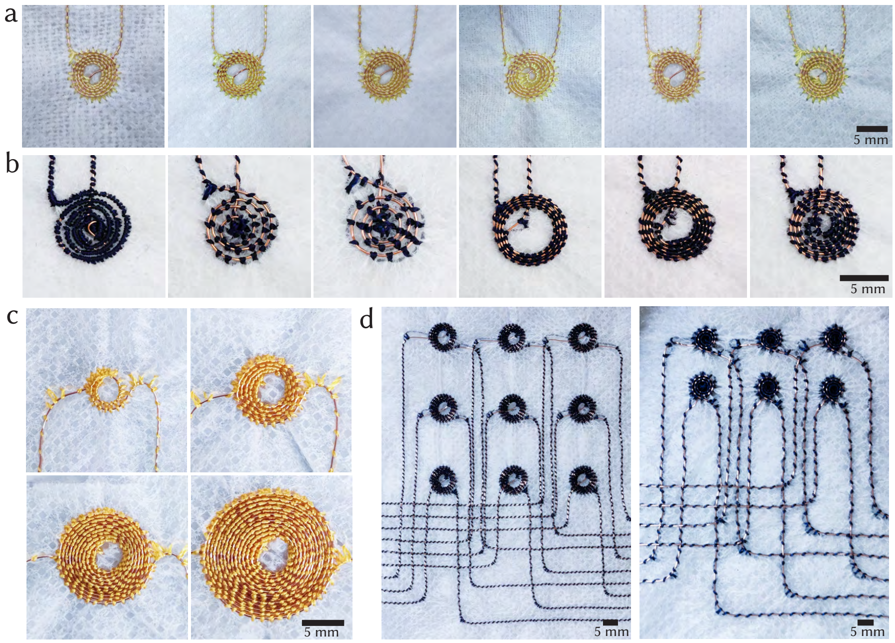

Supplementary Fig. 1: Embroidered vibrotactile coil designs. a Embroidered coil on canvas, silk, cotton, mixed polyester-cotton, polyester, and poly-cotton substrate. **b** Embroidered coil with various stitch pitch (p_s) and coil pitch (p_c). **c** Embroidered coils with 2, 5, 10, and 15 winds (outer radius of 3.25, 4, 5.25, 6.5 mm). **d** Embroidered coil arrays with different spacing and arrangements. Both ends of the coils are arranged to the side for electrical connections to the driving board.

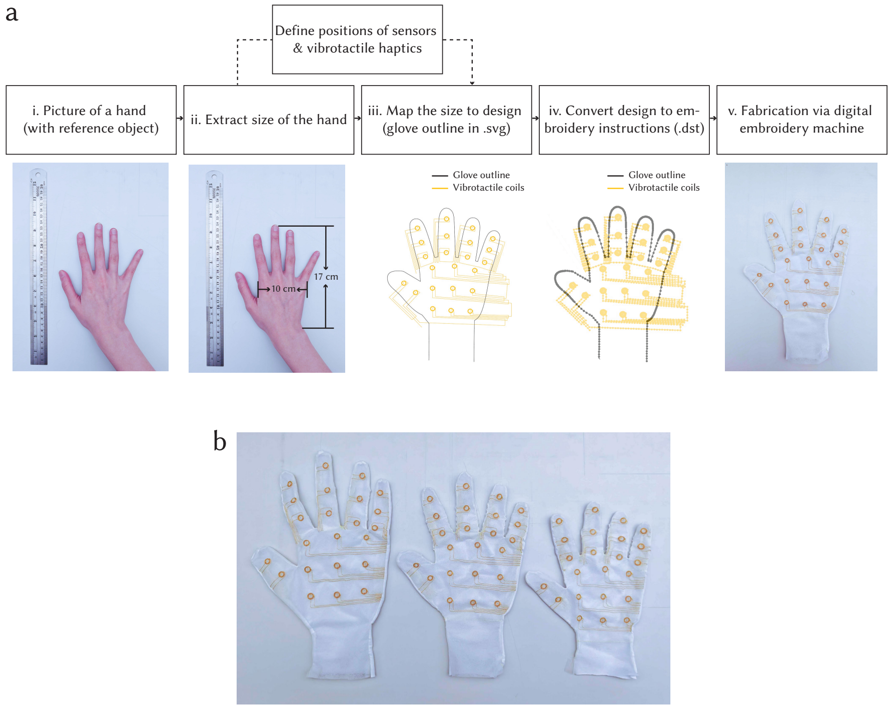

Supplementary Fig. 2: Smart gloves customization pipeline. a We first take a picture of a person's hand with a fixed reference (i). We then extract key parameters on the size of the hands from the pictures, such as the length of fingers, the width of the palm, and so on (ii). Such parameters were then able to be mapped to a pre-defined glove design template in any vector-based interactive user interface, e.g. Adobe Illustrator, Inkscape, etc (iii). The placement of tactile sensors and vibrotactile haptic units can be customized optionally. The customized glove designs were then converted into embroidery machine file and was automatically fabricated via the the machine (iv and v). **b** Photograph of fabricated gloves in small (12 cm x 23 cm), medium (11 cm x 20 cm), and large sizes (10 cm x 17 cm).

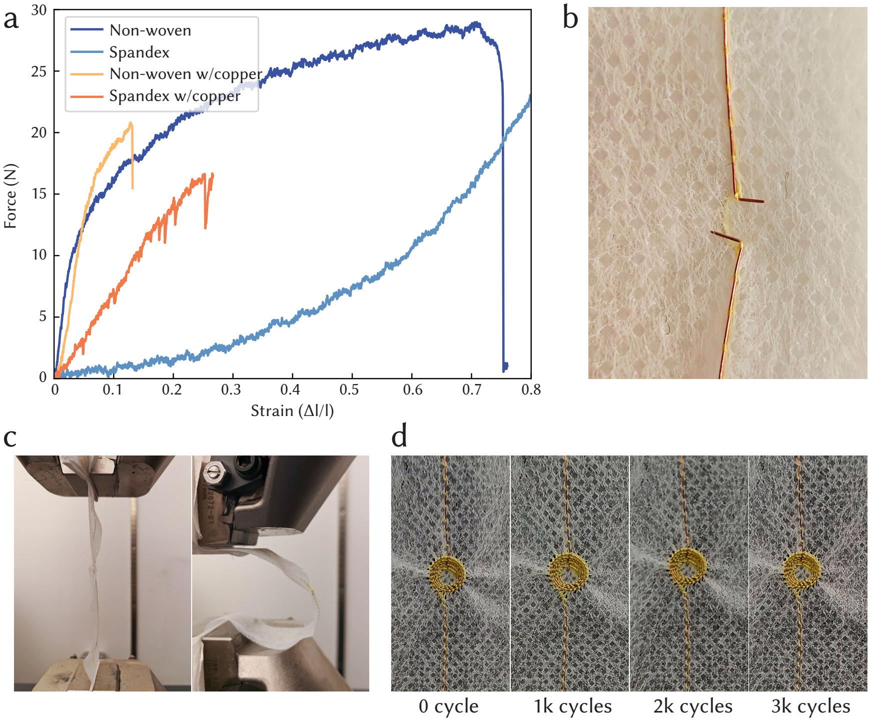

Supplementary Fig. 3: Mechanical characterization. a Tensile tests on pure non-woven and spandex fabric substrates as well as samples with embroidered embroidered vibrotactile coils. **b** The fracture of embroidered enameled copper wire is the limiting factor to the elongation of the presented textile sample. **c** Cyclic bending tests with the minimum radius of 3.18 cm. **d** Photographs of an embroidered vibrotactile coil and its connections after 0, 1000, 2000, and 3000 bending cycles. No changes were observed.

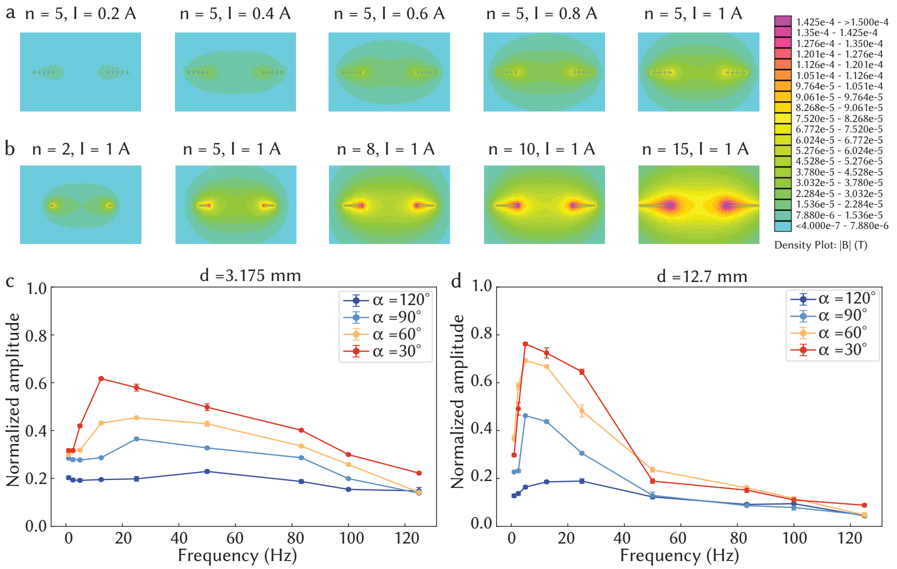

Supplementary Fig. 4: More simulation and characterization on the embroidered vibrotactile unit. a Simulation on the generated magnetic flux of the same coil design (32 AWG enameled copper wire, r_{in}=3 mm, p_c=0.5 mm, p_s=1 mm) with different input AC. **b** Simulation on the generated magnetic flux of embroidered coils with different numbers of winds (32 AWG enameled copper wire, r_{in}=3 mm, p_c=0.25 mm, p_s=1 mm) at the same input AC. **c** Normalized vibration displacement of haptic units of different radii (the radius of the tagged permanent magnet matches the outer radius of the embroidered coil r_{out}) with various pre-cut left-out slit angles (α). The trends indicate the resonant frequency of haptic units of different sizes and designs. Error bars indicate the standard deviations (SD) across measurements.

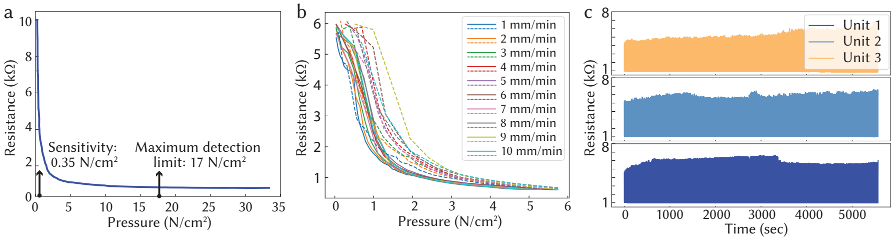

Supplementary Fig. 5: More tactile sensor characterization. a A typical tactile sensor obtains a minimum detection limit of 0.35 N/cm2 and a maximal detection limit of 20 N/cm^2. The linear region I and II obtain a sensitivity of 1000 $\Omega/(N/cm^2)$ and 25 $\Omega/(N/cm^2)$ respectively. **b** Resistance profile of typical tactile sensing unit under loading and unloading cycle at different speeds. The solid and dashed lines indicate the sensor performance during the pressure loading and unloading process respectively. **c** Three individual tactile sensors obtain similar and stable performance under 2000 loading and unloading cycles (0 - 5.5 N/cm^2).

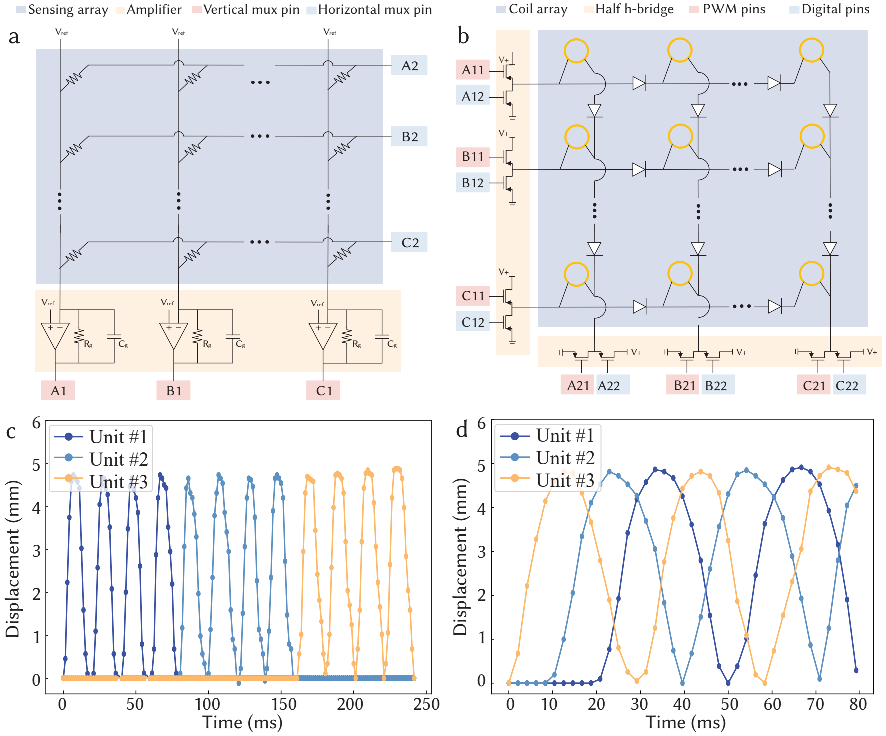

Supplementary Fig. 6: Sensing readout and vibrotactile driving circuit. a A modified electrical-grounding-based isolation circuit architecture for passive sensing array readout. **b** A matrix-based vibrotactile driving circuit. **c**, **d** Vibration displacement of neighboring haptic units when driven in a sequential and multiplexed manner.

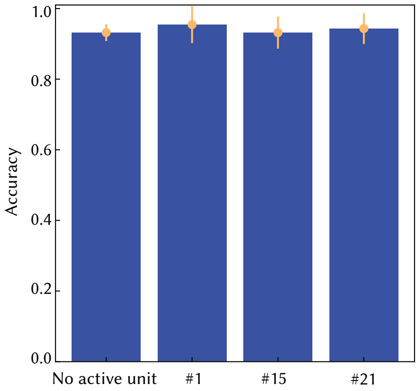

Supplementary Fig. 7: Consistency of user perception with neighboring vibrotactile interference. Users classify vibrotactile stimulus positions with similar accuracy when there is no interference and when an individual unit keeps vibrating as interference. Error bars indicate the standard deviations (SD) across measurements.

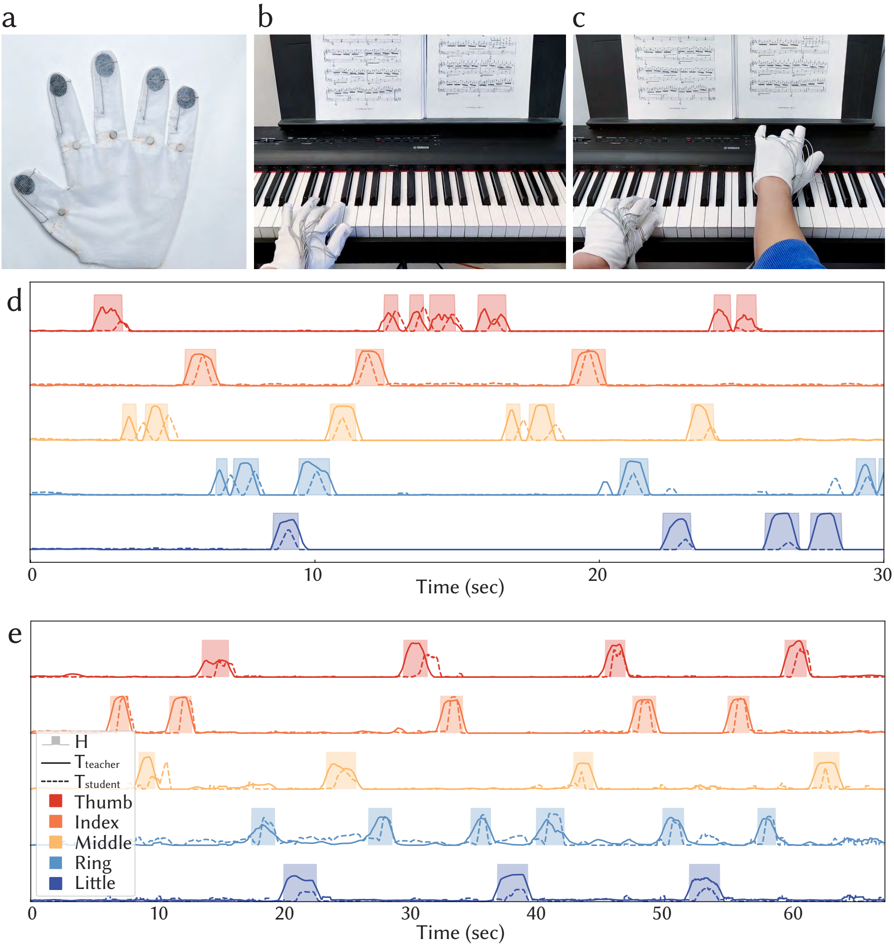

Supplementary Fig. 8: Transfer tactile interaction for piano instructions. a A full-sized tactile-haptic glove with tactile sensors at the fingertips and vibrotactile units on the inside of each finger. The experiment involves two users, one acting as a teacher and one acting as a student. The tactile sensors capture the pressure imprints from the teacher, which can be converted to the corresponding vibrotactile haptic feedback and guide the student for optimal sequence playing (**b**) offline, and (**c**) in real time. Qualitative results on the transferring of tactile interactions (**d**) offline, and (**e**) in real time.

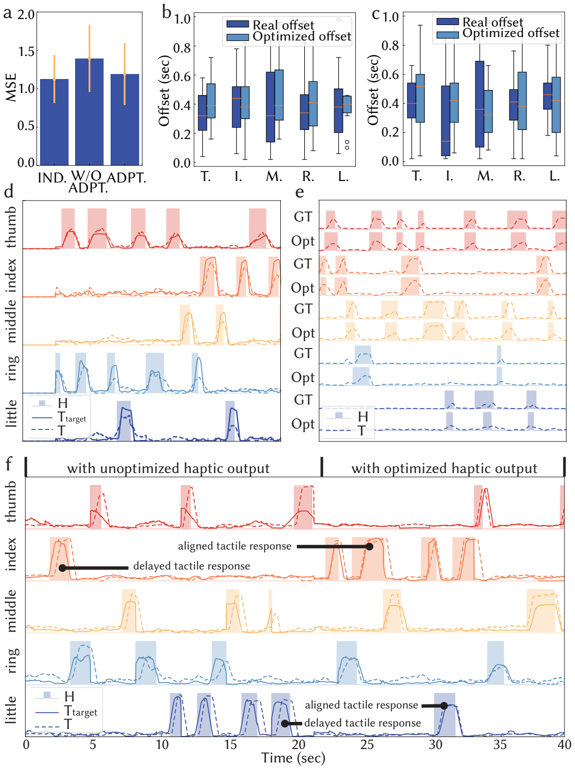

Supplementary Fig. 9: Offline validation of haptic output optimization. a Quantitative comparison of the accuracy of the individually-trained forward model (IND.), universal model without adaptation module (W/O ADPT.), and universal model with adaption model (ADPT.). **b** The forward dynamics model is able to predict the time offset for individual user's perception and reaction toward the haptic instructions. **c** The inverse optimization pipeline predicts the time offset of the vibrotactile haptic feedback for each individual user's optimal performance. The labels along the x-axis indicate five individual fingers. **d** Qualitative result of the forward dynamics model, where the predicted tactile response from the given haptic instructions aligns with the ground truth. **e** Offline qualitative evaluation of the haptic feedback inverse optimization, where the optimized haptic instructions optimized based on a given target tactile sequence align with the ground truth haptic instructions. **f** Online qualitative evaluation of the haptic instructions optimization pipeline. It is evident when the user is provided with unoptimized haptic instructions that there is a discrepancy between the target tactile sequence and the output tactile sequence (especially the time offset). Such discrepancy alleviates when our optimized haptic instructions is provided.

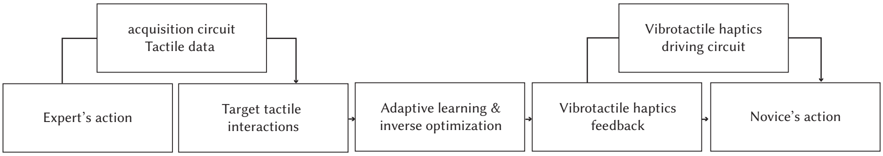

Supplementary Fig. 10: Tactile interactions transfer pipeline. We first extract the tactile interaction from the expert's action via the tactile sensor acquisition circuit. This tactile data is then fed into the adaptive learning and inverse optimization pipeline, which generates the optimized haptic sequence. The optimized haptic sequence is subsequently received and executed by the vibrotactile haptic driving circuit to provide real-time feedback for guiding the novice's actions.

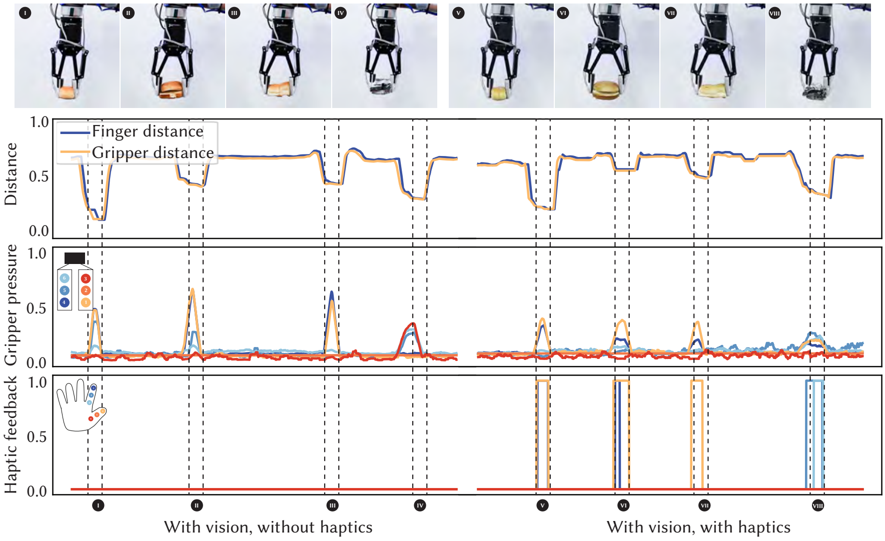

Supplementary Fig. 11: Tactile interaction transfer for teleoperation with visual feedback. Qualitative results on tele-grasping of a hot dog bun, burger bun, Hawaiian bread, soft plastic box with vision. Regardless of tactile interaction transfer, users were able to adjust the grasps based on visual information when visual feedback was provided. However, when both visual and haptic feedback was offered, users were able to extract information from the tactile information transferred by the tactile-haptic gloves and complete the grasp with relatively minimal pressure applied to the object (as shown in the gripper pressure plot).

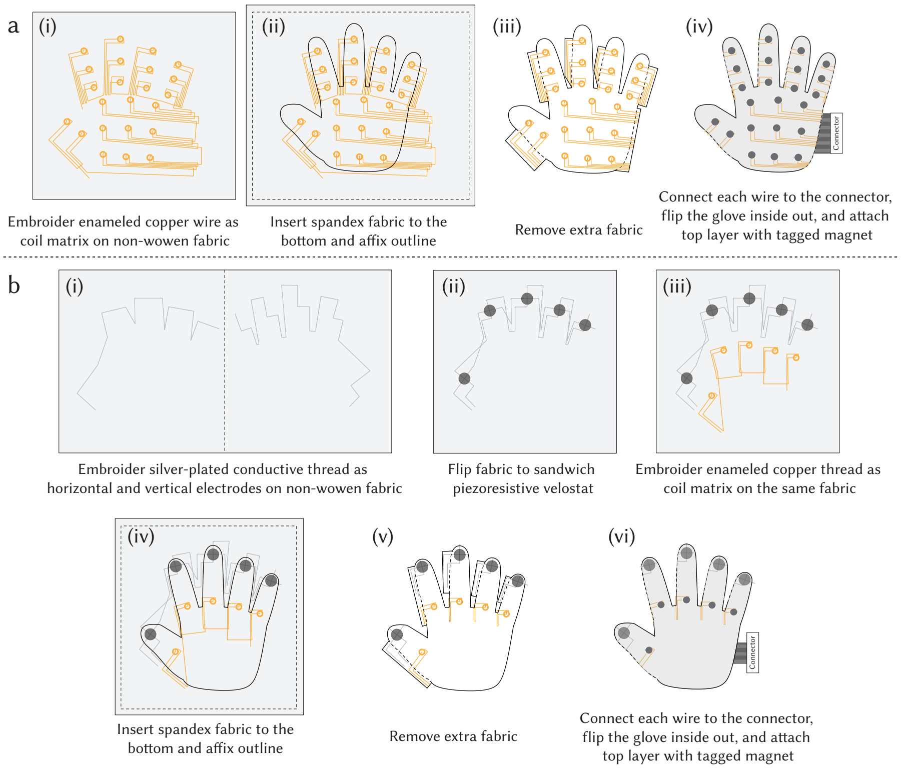

Supplementary Fig. 12: Fabrication. a Fabrication of a textile-based vibrotactile haptic glove consists of 4 steps. First, we embroider the magnetic wire into a coil array on a non-woven fabric substrate through a digital embroidery machine (i). Then, we insert a Spandex fabric to the bottom of the fabric substrate and stitch the outline of a full-sized glove (ii). The excess fabric is removed by laser cutting or hand cutting (iii). Lastly, we connect the electrodes to the diving board, flip the full-sized glove inside-out, and align and attach the top fabric layer with pre-cut slits and tagged permanent magnets (iv). **b** A full-sized glove with tactile sensing array and vibrotactile haptic array can be fabricated in sequential machine runs. First, we embroider the silver-plated thread as vertical and horizontal electrodes of the tactile sensing array (i). We then sandwich piezoresistive films with embroidered electrodes (ii). The same procedure for textile-based vibrotactile haptic array can be applied on the fabric with an integrated tactile sensing array (iii-vi).

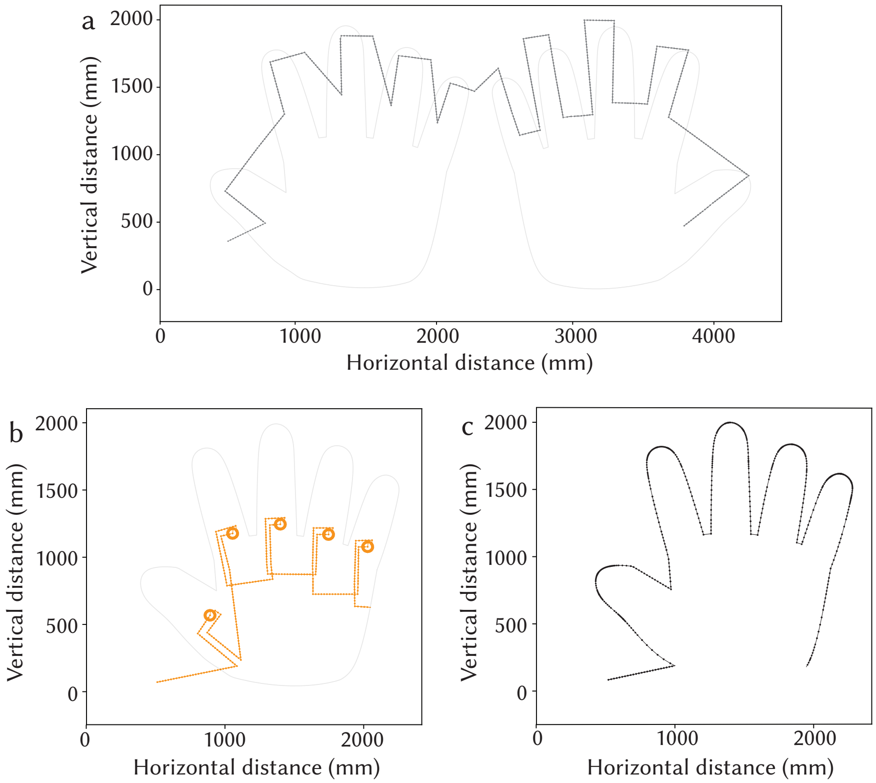

Supplementary Fig. 13: Embroidery designs for fabrication of a tactile-haptic glove. Designs of embroidered (**a**) tactile sensor electrodes, (**b**) magnetic coils (**c**) full-sized glove outline. Each dot represents an embroidery stitch. Units are in mm.

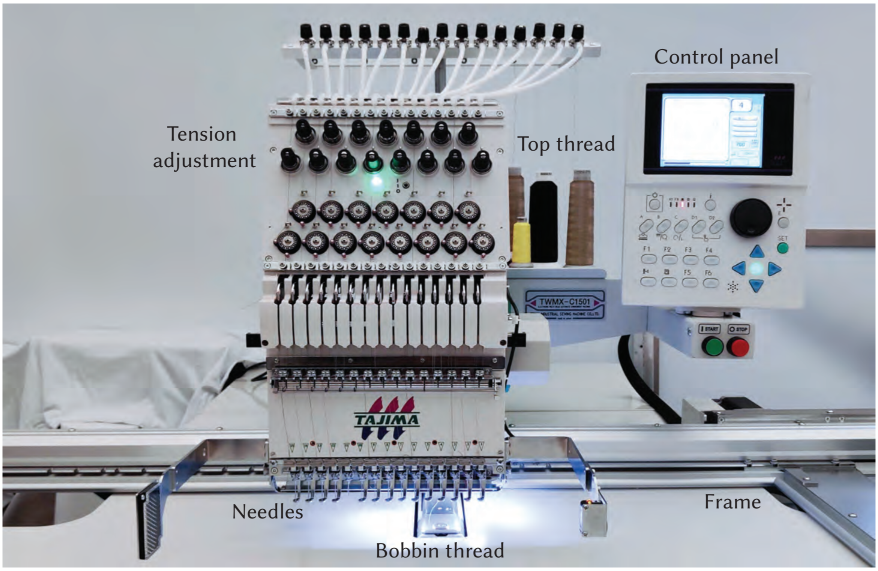

Supplementary Fig. 14: Digital embroidery machine. The Tajima TWMX-C1501 digital embroidery machine offers a 50 cm by 80 cm textile mounting frame, 15 top thread carriers with tension adjustment, and 1 bobbin loading space.

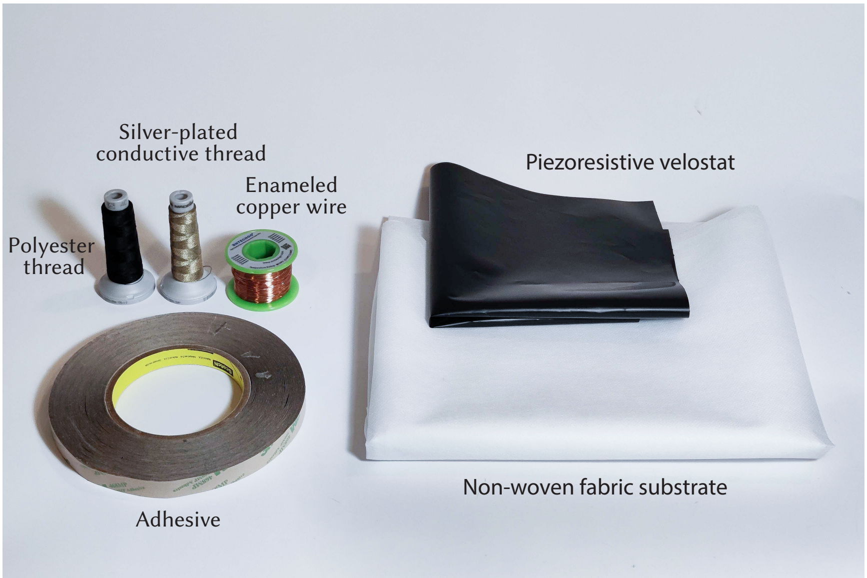

Supplementary Fig. 15: Fabrication materials. The tactile sensing array is made of silver-plated conductive thread, polyester thread, non-woven fabric, adhesive, and piezoresistive velostat film. The vibrotactile haptic array is made of enameled copper wire, polyester thread, adhesive, and non-woven fabric substrate.
