# Supplementary material for: Adaptive tactile interaction transfer via digitally embroidered smart gloves
Source: Nat Commun. 2024 Jan 29;15:868. doi: 10.1038/s41467-024-45059-8 (PMC10825181; doi:10.1038/s41467-024-45059-8)
Supplement: Supplementary file 3 — Description of Additional Supplementary Files [file 41467_2024_45059_MOESM3_ESM.pdf]

Description of Additional Supplementary Files

Supplementary Data

File Name: Supplementary Data 1
Description: Sample data and codes for the training of adaptive human behavior modeling and reverse haptics optimization.

Supplementary Movies

File Name: Supplementary Movie 1
Description: Fabrication of textile-based vibrotactile haptic unit by integrating enameled copper thread using a digital embroidery machine.

File Name: Supplementary Movie 2
Description: Resonant frequency of different embroidered vibrotactile haptic unit designs. Displacement of vibrotactile haptic units of different designs at different frequencies.

File Name: Supplementary Movie 3
Description: Visualization of textile-based vibrotactile haptic units vibration on a glove. The vibration of haptic units covered by reflective films on a glove.

File Name: Supplementary Movie 4
Description: Tactile interactions transfer within an individual user for tactile occlusion alleviation. Real-time tactile information was captured from the outer thick animal handling glove and haptic feedback was displayed on the inner vibrotactile glove.

File Name: Supplementary Movie 5
Description: Offline and real-time tactile interaction transfer across users for piano training.

File Name: Supplementary Movie 6
Description: Adaptive tactile interaction transfer across users for improved performance in rhythm gaming and car racing gaming. With our adaptive haptics optimization pipeline, the haptic feedback sequence displayed to the student was optimized based on user-specific behaviors and the tactile information captured from experts.

File Name: Supplementary Movie 7
Description: Tactile interaction transfer between human and robot for teleoperation. We demonstrate the teleoperation of a parallel gripper for deformable object grasping without visual or haptic feedback, without visual but with haptic feedback, with visual but without haptic feedback, and with both visual and haptic feedback.
